# Probiotic Potential of *Leuconostoc pseudomesenteroides* and *Lactobacillus* Strains Isolated From Yaks

**DOI:** 10.3389/fmicb.2018.02987

**Published:** 2018-12-04

**Authors:** Yaping Wang, Aoyun Li, Xiong Jiang, Hui Zhang, Khalid Mehmood, Lihong Zhang, Jinhuan Jiang, Muhammad Waqas, Mujahid Iqbal, Jiakui Li

**Affiliations:** ^1^College of Veterinary Medicine, Huazhong Agricultural University, Wuhan, China; ^2^Hubei Three Gorges Polytechnic, Yichang, China; ^3^University College of Veterinary and Animal Sciences, The Islamia University of Bahawalpur Pakistan, Bahawalpur, Pakistan; ^4^College of Animals Husbandry and Veterinary Medicine, Tibet Agriculture and Animal Husbandry University, Linzhi, China

**Keywords:** antibacterial activity, safety, *Lactobacillus* strains, *Leuconostoc pseudomesenteroides*, yaks

## Abstract

The purpose of this study was to evaluate the antibacterial activity and safety of bacterias with probiotic potential isolated from free-ranging Tibetan yaks in high altitude regions of Tibet. For this purpose, one *Leuconostoc pseudomesenteroides* strain (named P1) and two *Lactobacillus johnsonii* and *Lactobacillus mucosae* strains (named LY1 and LY2), respectively, were isolated from fecal samples of Tibetan yaks. The antibacterial activity of the isolates was studied using *Escherichia coli* (*E. coli* ATCC 25922), *Staphylococcus aureus* (*S. aureus* ATCC 26112), and *Salmonella enteritidis (S. enteritidis NCTC 13349)* as indicator pathogens. The results showed that LY1 had high antibacterial efficacy against *E. coli* and *S. enteritidis*, while P1 had the most powerful bacteriostatic ability against *S. aureus*. PCR amplification showed that all the isolated strains were positive for *Ent P2* (enterocin P-like bacteriocin) and exhibited a high tolerance to bile and low pH. Moreover, the safety of P1, LY1, and LY2 was determined through antibiotic resistance experiments, resistance gene testing, and hemolytic analysis while the antibacterial activity was assessed by *in vitro* and *in vivo* experiments. The LY2 strain was abandoned as a potential probiotic due to the detection of the *vanA* gene. The mice were fed from days 1 to 30 in six groups, the P1-1 (gavaged with P1 1 × 10^8^ CFU/day), P1-2 (gavaged with P1 1 × 10^9^ CFU/day), LY1-1 (gavaged with LY1 1 × 10^8^ CFU/day), LY1-2 (gavaged with LY1 1 × 10^9^ CFU/day), control (gavaged with an equal volume of vehicle), and blank control (gavaged with an equal volume of vehicle) groups. After 30 days, mice in the P1-1, P1-2, LY1-1, LY1-2, and control groups were intraperitoneal challenged with 1 × 10^8^ CFU of *E. coli* (*n* = 10) in the abdomen. After 2 days of infection, the mice in the control group showed more severe damage in the liver, spleen and intestine than the mice in the P1-2 and LY1-2 groups. The mice in the P1-2 and LY1-2 groups had lower rates of diarrhea and mortality than other groups. In conclusion, bacteria with probiotic potential isolated from yaks may possibly be effective and safe antibacterial substances, providing a new treatment method to reduce the incidence of diarrhea associated with bacterial diseases in yaks.

## Introduction

Infectious diseases are a threat for animal health and productivity in developing countries (Elhaig et al., 2016; Wen et al., 2016; Yilmaz et al., 2016; Mehmood et al., 2017). Diarrhea is a common problem in cattle that greatly affects the growth of animals and causes huge economic losses (Heuer et al., 2007). Diarrhea can be caused by many factors, such as bacteria and viruses. Many measures have been carried out to prevent diarrhea, including improvements in hygiene and feeding management systems, but it still occurs from time to time (Cho et al., 2013). *Escherichia coli* (*E. coli*), *Salmonella enteritidis* (*S. enteritidis*), and *Staphylococcus aureus* (*S. aureus*) are considered common pathogens that cause diarrhea (Dong et al., 2018). Antibiotics are widely used to treat diarrhea, but antibiotic use is considered a double-edged sword, as some problems have emerged with antibiotic usage, especially antibiotic resistance. Previous studies have shown that the misuse of antibiotics has become a serious problem, affecting microbial balance and increasing the incidence of antibiotic-associated diarrhea by 5 to 25% (Kaltenbach and Heitz, 2004). Probiotics are living microorganisms that not only improve the balance of intestinal microflora but also inhibit many pathogens (Food Agriculture Organization/World Health Organization [FAO/WHO], 2011). They are widely used in animal production because of their antibacterial activity and various biological characteristics. Some researchers have reported that *Lactobacillus johnsonii* BS15 (CCTCC M2013663) can be used to prevent non-alcoholic fatty liver disease in obese mice and improve growth performance along with meat quality (Wang H. et al., 2018). In addition, some studies have shown that *L. johnsonii* can improve lipid metabolism and intestinal microflora in chickens to prevent subclinical necrotic enteritis and reduce respiratory viral infections (Fonseca et al., 2017). Moreover, it has been reported that *Leuconostoc mesenteroides* can inhibit the growth of pathogens and can be used as a safe probiotic for further study (Makhloufi et al., 2013; Casado et al., 2014). However, some cases of bacteremia, liver damage and other clinical symptoms may be related to some *Lactobacillus*). Screening strains, as per previous reports (Rautio et al., 1999). The oral toxicity test in animals has been proposed as the basis for evaluating the safety of probiotics (Stine and Brown, 1996) for edible probiotics requires the following steps: (1) identifying the bacteria, (2) testing for probiotic potential through *in vitro* experiments, (3) proving that the probiotics are safe and effective, and (4) performing experiments in animals (Food Agriculture Organization/World Health Organization [FAO/WHO], 2006).

Yaks are long-haired bovines that live on high plateaus and in anoxic zones (Zhang et al., 2018; Wu et al., 2018a,b). Approximately 90% of the global yak population resides in the Qinghai, Tibet and Sichuan provinces of China (Li et al., 2015). Yaks are an important source of milk, meat and leather for local herders. However, it has been reported that diarrhea occurs in Tibetan yak calves. Therefore, the present study was designed to isolate bacteria with probiotic potential that can effectively inhibit diarrhea and reduce various losses associated with diarrhea in yaks.

In this study, the antibacterial activity of strains isolated from the feces of healthy Tibetan yaks was assessed with *in vitro* and *in vivo* experiments. The safety of the strains was evaluated with drug resistance experiments, drug resistance gene testing, hemolytic analysis and oral toxicity testing in mice. The purpose of this trial was to assess the safety of the isolated strains and to assess whether they can reduce the incidence of bacterial diseases in a mouse model. The results of this study may provide new ideas and agents for the treatment of diarrhea associated with bacterial diseases in yaks.

## Materials and Methods

### Sample Collection

A total of 30 fecal samples were randomly collected from different ages and genders of free-ranging yaks in the Nyingchi region of Tibet, China. The samples were kept at 4°C and transported to Huazhong Agricultural University, Wuhan, and they were then stored at -80°C for further experiments. In addition, *E. coli, S. enteritidis*, and *S. aureus* were provided by the Veterinary Science Center of Huazhong Agricultural University, China.

### Isolation and Identification of *Lactobacillus* Strains

Three strains were isolated from the feces collected from healthy free-ranging Tibetan yaks. A portion (2 g) of each sample was blended in phosphate-buffered saline and shaken well for 30 min at 37°C. The supernatant was streaked on de Man, Rogosa and Sharpe (MRS, a selective agar for lactic acid bacteria) agar (Hangzhou Reagents, China) and then grown for 48 h at 37°C in an incubator under anaerobic conditions. Milky white and raised suspected lactic acid bacteria colonies were selected for purification and cultivation for three generations and then individual colonies were picked and inoculated in MRS broth for culture. For further validation, milky white raised colonies were tested by Gram staining and subjected to biochemical analysis. Finally, all isolated strains were confirmed and identified by genetic analysis using PCR and 16S rRNA sequencing. The genomic DNA of the *Lactobacillus* strains (2 mL *Lactobacillus* culture, 1 × 10^8^ CFU/mL) was extracted using a Bacterial DNA Isolation Kit (Foregene, China). The universal PCR primers 27F (5′-AGAGTTTGATCCTGGCTCAG-3′) and 1492R (5′-TACGGCTACCTTGTTACGACTT-3′) were used to amplify the 16S rRNA gene (Wang L. et al., 2018). Furthermore, the PCR products were sequenced by the Qingke Biotech Company (Wuhan, China) and subjected to BLAST analysis on the NCBI website. The phylogenic tree was constructed using the neighbor-joining method with MEGA 6 software.

### Antibiotic Susceptibility Assay

The drug sensitivity of isolated strains was tested with the disk diffusion method (Ghosh et al., 2015). The isolated strains and indicator strains (1 × 10^8^ CFU/mL) were aspirated onto MRS agar plates and Luria-Bertani (LB) agar plates. The inhibition zone diameter was measured after 24 h of incubation at 37°C by using 12 drugs-sensitive tablets (vancomycin 30 μg, norfloxacin 10 μg, teicoplanin 30 μg, ciprofloxacin 5 μg, amoxicillin 25 μg, roxithromycin 15 μg, cephalexin 30 μg, lincomycin 2 μg, tetracycline 30 μg, erythromycin 15 μg, ceftriaxone 30 μg, and enrofloxacin 10 μg) from Hangwei Biotechnology Company, China (Mandal et al., 2016). Each antibiotic was graded as resistant (R), intermediate (I) or sensible (S) according to National Committee for Clinical Laboratory Standards (NCCLS) (National Committee for Clinical Laboratory Standards [NCCLS], 2003).

### Acid and Bile Tolerance Properties

A good potential probiotic needs to be stable at a low pH and in the presence of high bile salts in the stomach and digestive tract (Khalil et al., 2018). Therefore, we carried out acid and bile resistance experiments using the Kobierecka’s method, with a few improvements (Kobierecka et al., 2017). Bacterial solutions (1 mL, 1 × 10^8^ CFU/mL) were inoculated into MRS broth (7 mL) and incubated at 37°C. The survival rate was calculated with the viable plate counting method after 3 h. The MRS broth was adjusted to pH 2, pH 3, pH 4, and pH 5 with 1 M HCl, and the control broth was broth without HCl. To determine the survival rate of the strains under different bile salt concentrations, the strains were cultured for 24 h and spread evenly on MRS agar plates with bile salt concentrations of 0.1, 0.2, 0.3, 0.4, and 0.5% ox gall. Non-salted MRS agar plates served as controls. After 48 h, the viable colonies were counted.

### *In vitro* Antibacterial Tests

The inhibition of the growth of pathogens is an important feature of probiotics (Khalil et al., 2018). The ability of the isolated strains to resist pathogenic bacteria was determined with an agar diffusion test (Zhang et al., 2016). *E. coli* (1 × 10^8^ CFU/mL), *S. enteritidis* (1 × 10^8^ CFU/mL) and *S. aureus* (1 × 10^8^ CFU/mL) were used as the indicator pathogens in this experiment. The isolated strains were incubated at 37°C for 24 h in MRS broth and then 100 μL (1 × 10^8^ CFU/mL) of bacterial solution was injected into LB agar plates punched with a 5 mm punch. The inhibition zone diameter was measured after 24 h.

### Hemolytic Activity

All strains were cultured in MRS broth for 24 h, and then the bacterial solutions were streaked onto blood agar plates containing yak blood. The hemolytic activity of the strains was evaluated after 24 h at 37°C, and *S. aureus* was used as a positive control.

### Determination of Growth Performance of Isolated Strains

All isolated strains were cultured in MRS broth for 24 h, and then the bacterial solutions (1 mL, 1 × 10^8^ CFU/mL) were inoculated into MRS broth (7 mL). The OD_600_, CFU and pH were measured every 2 h up to 36 h (*n* = 3).

### PCR Amplification and Detection of the Bacteriocin Gene

The DNA sequences of isolated strains were used as templates, and *Ent P2* gene primers were used to detect the bacteriocin gene by PCR amplification as described by Kang and Lee (2005). The primer sequences for the *Ent P2* gene were F (5′-GCTACGCGTTCATATGGTAATGGTG-3′) and R (5′-ATGTCCCATACCTGCCAAACCAGAAGC-3′). The primers were synthesized by Qingke Biotechnology Company (Wuhan, China). The PCR conditions were similar to Kang and Lee (2005), except that the annealing temperature was changed to 60°C.

### PCR Detection of Antibiotic Resistance Genes

To identify the antibiotic resistance determinants, all the isolated strains were examined for the presence of tetracycline resistance genes [*tet (L), tet (M), tet (O)*, and *tet (S)*] (Aarestrup et al., 2000) and glycopeptide-resistant genes [*vanA* (Dutkamalen et al., 1995) and *vanB* (Ramos-Trujillo et al., 2003)]. For detection of the possible presence of integron, a class 1 integrase-specific fragment of the *Int1* gene was used (Sáenz et al., 2004). All the primer sequences used during the study are given in Supplementary Table S1.

### Animal Toxicity Study

Mice were selected as experimental animals to evaluate the safety of the isolated strains. Briefly, a total of 20 male and 20 female KM mice (15–18 g) were housed under standard hygienic conditions at a temperature of 22 ± 2°C and 55 ± 2% humidity with a normal light-dark cycle. The animals were fed a basal diet and had free access to water during the entire experimental period. After 3 days of acclimation, the mice were randomly divided into 4 equal groups (*n* = 10). Mice in three of the experimental groups received P1, LY1, or LY2 at 1 × 10^9^ CFU/day for 18 consecutive days. Mice in the control group were forcibly gavaged with an equal volume of vehicle. All animal protocols were carried out according to standard guidelines from the Good Laboratory Practice Guidelines (Hawkins, 1993). All the experiments were approved and reviewed by Institutional Animal Welfare and Research, Ethics Committee guidelines of Huazhong Agricultural University, Wuhan, China. The animals’ activity, behavior, hair luster, food intake, body weight and general health status were observed on a daily basis throughout the experiment. After 18 days of probiotic gavage, all the animals were sacrificed by cervical dislocation, and blood, liver and kidney samples were collected under sterile conditions. Statistical analysis of the data for multiple comparisons was performed by one-way analysis of variance followed by Duncan’s test. A level of *P* < 0.05 was considered statistically significant.

### Bacterial Translocation

Bacterial translocation was analyzed in the blood, liver and kidneys (Zhou et al., 2000). Approximately 50 μL of blood was inoculated onto MRS agar plates and brain-heart infusion (BHI) agar plates for 48 h at 37°C. Tissue samples were homogenized in buffered peptone water (1 g/mL), and 100 mL of homogenates was cultured on MRS and BHI agar plates under the same conditions as those for the blood. The translocation rate was calculated as follows: translocation rate of bacteria = number of mice in which translocation was detected/total number of mice. Positive growth on agar plates was defined by a colony count of at least one. A chi-square test was used to analyze differences between rates (bacterial translocation incidence).

### Preventive Effects of the Strains Against Bacterial Diseases in Mice

To evaluate the effects of the strains on preventing bacterial diseases, a total of 30 male and 30 female KM mice (15–18 g) were housed under the same conditions as those described previously. The mice were assigned to 6 treatment groups, including the P1-1 (gavaged with P1 1 × 10^8^ CFU/day), P1-2 (gavaged with P1 1 × 10^9^ CFU/day), LY1-1 (gavaged with LY1 1 × 10^8^ CFU/day), LY1-2 (gavaged with LY1 1 × 10^9^ CFU/day), control (gavaged with an equal volume of vehicle), and blank control (gavaged with an equal volume of vehicle) groups. After 30 days, mice in the P1-1, P1-2, LY1-1, LY1-2, and control groups were intraperitoneal challenged with 1 × 10^8^ CFU of *E. coli* in the abdominal region, while the mice in blank control group were intra-peritoneal challenged with an equal volume of vehicle. After 2 days of infection, the surviving animals were sacrificed via cervical dislocation, and the liver, duodenum and spleen were harvested. Small specimens of the liver, spleen and duodenum were placed in 10% neutral formalin and routinely processed by embedding in paraffin. Tissue sections (4–5 μm) were stained with hematoxylin and eosin and examined under a light microscope. The animals’ activity, behavior, death, diarrhea, and general health status were closely observed throughout the experimental period. The presence of diarrhea was observed as mucoid, watery soft stools, often with a wet area around the anus according to Bolick et al. (2014).

## Results

### Isolation and Identification of Potential Probiotics

**FIGURE 1:**
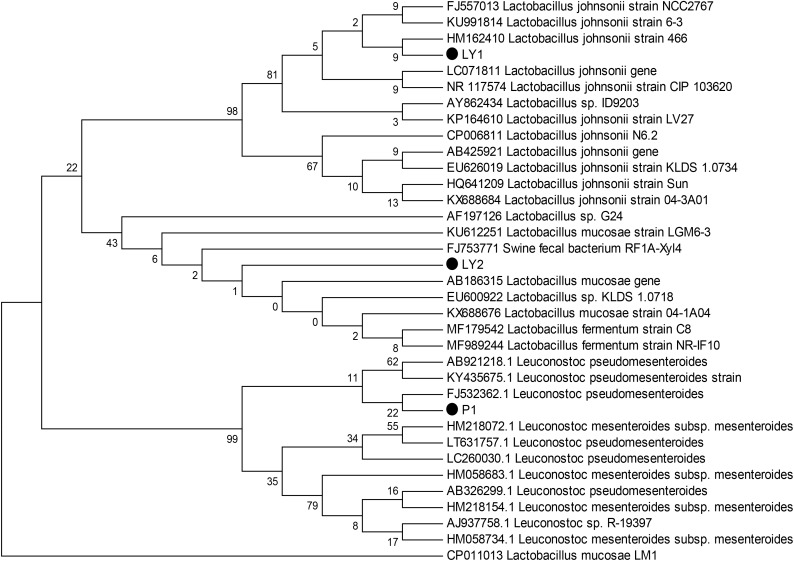
Phylogenetic tree based on the 16S rRNA gene sequences using the neighbor-joining method. The three isolated strains were P1, LY1, and LY2. The phylogenetic tree was statistically evaluated using 1000 bootstrap replicates.

We observed milky white and raised colonies on MRS agar and three strains were selected: P1, LY1, and LY2. All strains were Gram-positive and catalase-negative. In addition, 16S rRNA sequence analysis showed that P1 was 99% homologous to *Leuconostoc pseudomesenteroides*, LY1 was 99% homologous to *L. johnsonii* and LY2 was 99% homologous to *Lactobacillus mucosae.* The phylogenic tree was constructed using the neighbor-joining method with MEGA 6 software (Figure 1).

### Antibiotic Susceptibility Assay

P1, LY1, and LY2 showed a low degree of drug resistance (Table 1). LY1 was sensitive to all the antibiotics used in this study. Resistance rates of P1 and LY2 were lower than *S. aureus* and *S. enteritidis* as expected.

**Table 1:** Antibiotic susceptibility test for P1, LY1, LY2, and the standard indicator strains.

Antibiotics	P1	LY1	LY2	*E. coli*	*S. aureus*	*S. enteritidis*
Vancomycin	R	S	R	R	S	S
Norfloxacin	R	S	R	S	R	R
Teicoplanin	R	S	R	S	S	S
Ciprofloxacin	S	S	R	S	R	R
Amoxicillin	S	S	S	R	R	S
Roxithromycin	S	S	S	S	R	R
Cephalexin	R	S	S	S	I	R
Lincomycin	S	S	S	R	R	R
Tetracycline	S	S	S	S	S	S
Erythromycin	S	S	S	R	R	R
Ceftriaxone	R	S	S	S	R	R
Enrofloxacin	S	S	S	S	S	S

*S, sensitive; R, resistant; I, intermediate. The test result was based on NCCLS*.

### Acid and Bile Salts Tolerance

**FIGURE 2:**
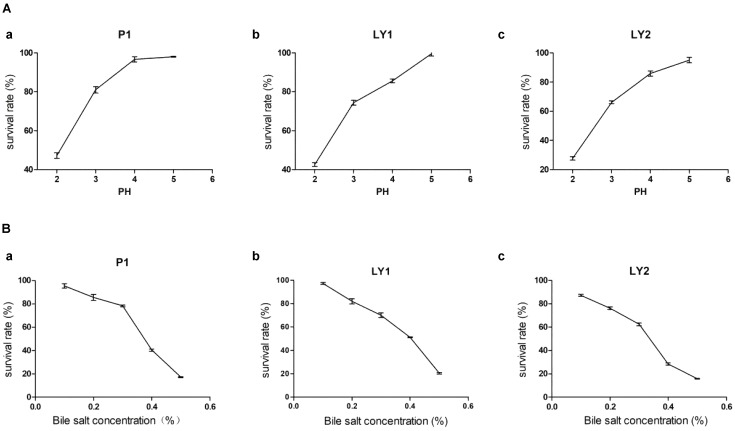
Acid and bile salts tolerance. **(A)** The tolerance of the isolated strains to acid. **(a)** P1. **(b)** LY1. **(c)** LY2. **(B)** The tolerance of the isolated strains to bile salts. **(a)** P1. **(b)** LY1. **(c)** LY2.

The isolates were incubated at pH 2, 3, 4, and 5 and at bile salt concentrations of 0.1, 0.2, 0.3, 0.4, and 0.5% at 37°C for 3 h. Strain P1 appeared to be tolerant to a pH of as low as 4 but then began to lose survival while strains LY1 and LY2 began to lose viability at pH < 5. On the other hand, the three strains’ survival rates ranged from 60.43 to 97.40% when less than 0.3% bile salt concentration (Figure 2). The survival rates were calculated as follows: survival rate = [c/co] ^∗^ 100%, where c and co represent the number of colonies in the experimental group and the control group, respectively.

### Antibacterial Tests *in vitro*

**FIGURE 3:**
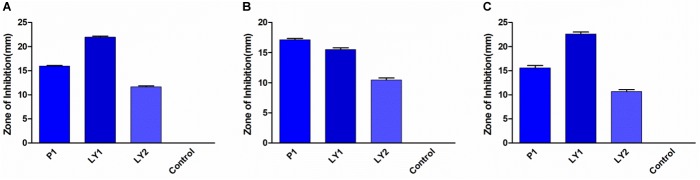
The results of the antibacterial experiment. **(A)** The inhibition zone diameters of P1, LY1, and LY2 against *E. coli*. **(B)** The inhibition zone diameters of P1, LY1, and LY2 against *S. aureus*. **(C)** The inhibition zone diameters of P1, LY1, and LY2 against *S. enteritidis*.

**FIGURE 4:**
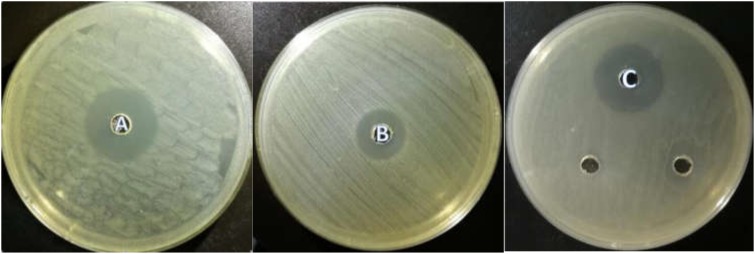
**(A)** The inhibition zone diameter of LY1 against *E. coli*. **(B)** The inhibition zone diameter of P1 against *S. aureus*. **(C)** The inhibition zone diameter of LY1 against *S. enteritidis.*

All the isolated strains showed inhibition activity against *E. coli, S. aureus*, and *S. enteritidis* in the well diffusion assay (Figure 3). The inhibition zone diameter ranged from 17.17 to 22.60 mm. LY1 exhibited greater inhibition of *E. coli*, with a diameter of 21.97 mm (Figure 4A). P1 showed the strongest inhibitory effect against *S. aureus*, with a diameter of 17.17 mm (Figure 4B). LY1 had the strongest inhibitory effect on *S. enteritidis*, with a zone of inhibition of 22.60 mm (Figure 4C).

### Isolated Strains Growth Time Curve

**FIGURE 5:**
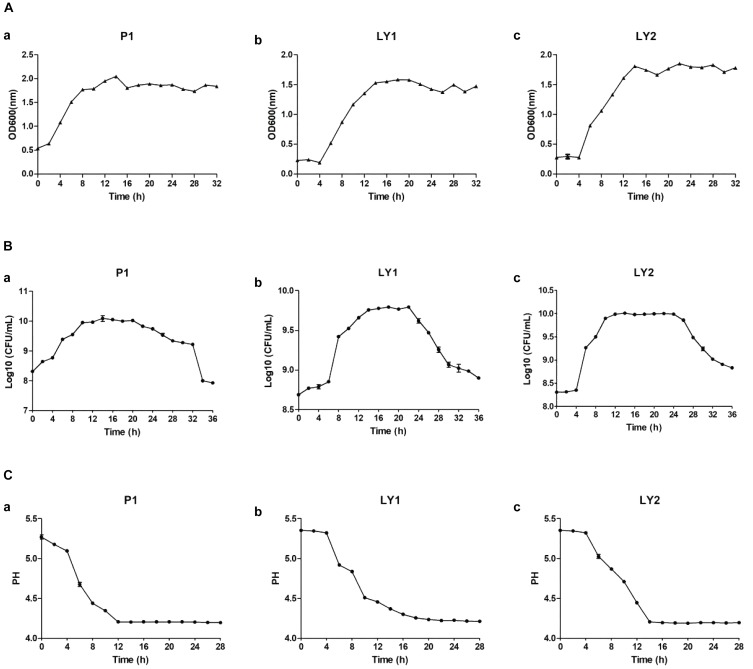
Measurement of OD_600_ values, colony-forming units and pH. **(A)** OD_600_ values measured every 2 h for a total of 32 h. **(a)** P1. **(b)** LY1. **(c)** LY2. **(B)** The colony-forming units were analyzed every 2 h for a total of 32 h, and the number of colonies was calculated as the log_10_ value of CFU per milliliter. **(a)** P1. **(b)** LY1. **(c)** LY2. **(C)** pH measurements taken every 2 h for a total of 26 h. **(a)** P1. **(b)** LY1. **(c)** LY2.

The results about isolated strains growth time curve are shown in Figures 5A–C. The growth of microorganisms in liquid cultures assayed by OD_600_ clearly showed that the logarithmic growth phases of P1 and LY1 were 2–12 and 4–14 h, respectively. At the same time, the pH values for P1 and LY1 clearly dropped. The CFU of P1 began to decrease after 24 h, and the OD_600_ value did not decrease. In the logarithmic phase of LY2, the pH value rapidly decreased at approximately 4–12 h, entered the plateau phase, and ultimately declined after 24 h, but LY1 entered the decline stage approximately 22 h later.

### Hemolytic Activity

After 24 h, the hemolytic activity of the strains was judged by observing the hemolytic rings on blood agar plates. Complete lysis (β-hemolysis) was caused by the positive control strain, whereas P1, LY1, and LY2 were not involved in the lysis of erythrocytes (γ-hemolysis).

### PCR Amplification of the Bacteriocin Gene

**FIGURE 6:**
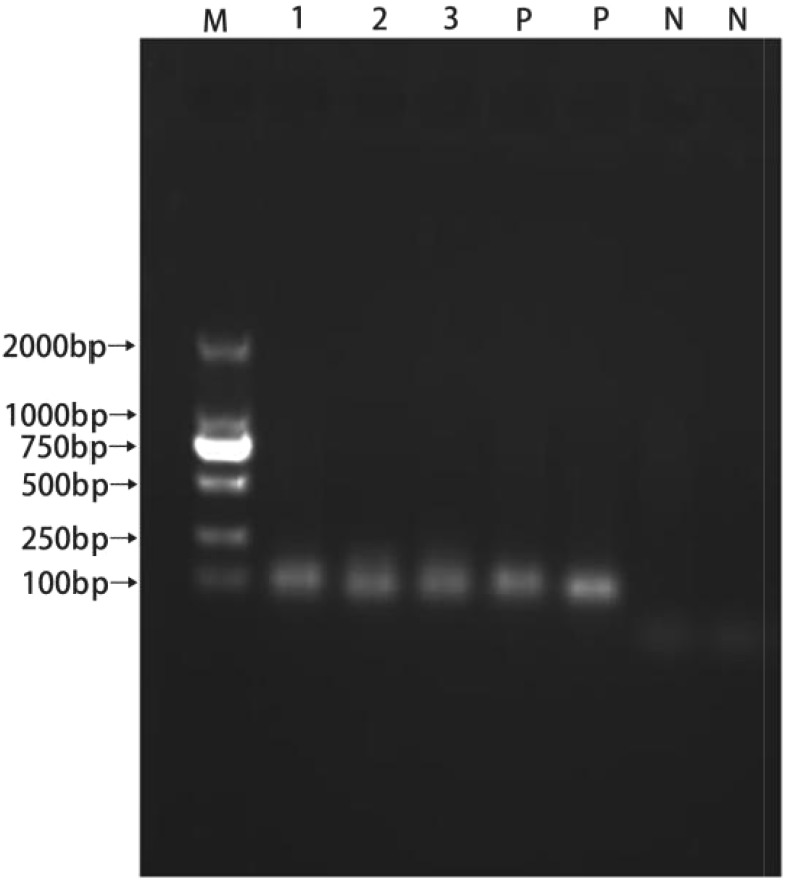
PCR products resulting from amplification with the enterocin P2 primers. (1) P1; (2) LY; (3) LY2; (P) positive control; (N) negative control.

All isolated strains were positive for *Ent P2*, and the template DNA and *Ent P2* primers produced amplicons of approximately 130 bp (Figure 6).

### Presence of Resistance Genes Analyzed by PCR

The tetracycline resistance-determining genes *tet (L), tet (S), tet (O)*, and *tet (M)* were not found in any of the isolated strains, and the strains did not contain the integron *Int1* gene. It was found that LY2 contained the *vanA* gene, but none of the strains had the *vanB* gene.

### Animal Toxicity Study

**FIGURE 7:**
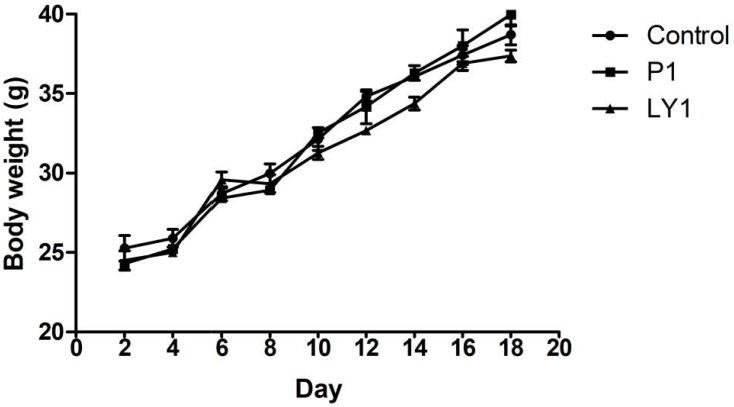
Body weight changes in the mice of the control and probiotic-treated groups. ● Represent the changes of body weight in the mice of the control group; ■ represent the changes of body weight in the mice of the P1 group; ▲ represent the changes of body weight in the mice of the LY1 group. The values are presented as the mean ± SD (*n* = 10). A level of *P* < 0.05 was considered statistically significant.

During the experiment, no animals were recorded to have symptoms such as disease, death or diarrhea. Animals in P1, LY1 and control groups did not show significant differences in behavior, hair, mental status or feed intake (data not shown). However, the data in LY2 group was abandoned because mice exhibited lethargy and unresponsiveness. The appearance of the internal organs and intestinal segments was normal, and no ulcers or adhesions were observed by macroscopic evaluation. As shown in Figure 7, there was no significant difference in the body weight changes between the control, P1 and LY1 groups.

### Translocation Rates of Bacteria

There was no statistically difference in the incidence of translocation of bacteria to the liver or kidney between the control group and any of the experimental, and no bacteremia was observed in any of the groups.

### Strains Preventing Bacterial Diseases

The mice in the control group showed the most serious disease symptoms: matted hair, poor mental state, high rate of diarrhea and death. Compared with mice in the control, P1-1 and LY1-1 groups, mice in the P1-2 and LY1-2 groups showed the lowest rates of diarrhea and mortality (Table 2). In this experiment, clear degeneration and congestion were detected within the livers of mice in the control group but not within those of mice in the blank control group. Mice in the P1-2 and LY1-2 groups exhibited reduced degeneration and congestion (Supplementary Figure S1). In terms of the spleen, mice in the control group showed severe lymphopenia, a large increase in red blood cells and a loss of normal structure compared with mice in the blank control group. The mice in the P1-2 and LY1-2 groups showed significant recovery compared to the mice in the control group (Supplementary Figure S2). In addition, degeneration was observed in the intestinal villi of the duodenum in the control group mice, whereas there was no degeneration in the P1-2 and LY1-2 group mice (Supplementary Figure S3).

**Table 2:** Performance of different groups of mice after infection with *E. coli*.

Virulence trait (s)	Death rate	Diarrhea rate
Blank control	0	0
Control	20%	60%
P1-1	0	30%
P1-2	0	20%
LY1-1	0	30%
LY1-2	0	10%

*5 Males and 5 females in each group*.

## Discussion

At present, antibiotics are widely used in the animal husbandry, humans, agriculture and aquaculture, but some problems have also emerged, especially antibiotic resistance (Tenover and Hughes, 1996). Diarrhea is a complex disease that can easily be caused by changes in the environment or other unfavorable conditions, and it is one of the major causes of economic losses in the cattle industry. *E. coli, S. enteritidis*, and *S. aureus* are common pathogens causing diarrhea (Yongil and Kyoungjin, 2014). Probiotics can maintain the balance of the intestinal flora, inhibit the colonization and survival of pathogens in the intestine and prevent inflammation (Archambaud et al., 2012; Yu et al., 2015). The purpose of our study was to find a safe and effective way to reduce the incidence of bacterial diseases in yaks. Recently, a large number of yaks have died due to bacterial infections. Yak meat and milk are necessities of daily life for Tibetans, so the use of antibiotics may pose a health risk to local herdsmen. Hence, the present study could be used as a reference for the prevention and control of bacterial infection in the Tibetan Plateau of China (Wu et al., 2017). The results show that LY1 has a strong antagonistic effect against *E. coli* and *S. enteritidis.* However, P1 had a strong antagonistic effect against *S. aureus*.

Bactericidal peptides can be produced by enterocins, including enterocins A, B, P, and L50, which are class II bacteriocins (Aymerich et al., 1996). Enterocin P is unique with a wide inhibitory spectrum (Herranz et al., 2001). In this study, all the isolated strains were positive for the *Ent P2* gene and showed positive antagonism against pathogenic bacteria. The key for probiotics to play a role in promoting the physiological health of the host is the ability to survive the intestine (Kaushik et al., 2009). Therefore, the isolated strain must be able to survive under low pH and high bile salt concentrations (Mandal et al., 2016). Isolated strains were tested for acidity and bile salt tolerance, and the results showed that they were highly tolerant, especially P1 and LY1.

According to the FAO/WHO report, although *Lactobacillus* is generally considered safe, they may cause some side effects (Food Agriculture Organization/World Health Organization [FAO/WHO], 2002). Therefore, it is recommended that antibiotic resistance and toxicity studies be used for safety testing. According to reports, some probiotics may cause hemolysis (Spinosa, 2009). The hemolytic properties of the isolated strains were tested, and no hemolytic activity was recorded for P1, LY1, or LY2. As a health threat, antibiotic resistance genes may be transmitted to bacteria in the gut by probiotics. Therefore, detection of the antibiotic resistance of probiotics is considered to be an important part of the safety testing of probiotics *in vitro* (European Food Safety Authority [EFSA], 2008). It has been reported that the *tet* gene present in some *Lactobacillus* strains may be transferred to other bacteria (Toomey et al., 2009). In the detection of resistance genes, the *van* gene deserves attention because vancomycin can be the last antibiotic used to cure infections caused by bacteria that are resistant to antibiotics (Woodford et al., 1995). In this study, the resistance test of the isolated strains and the indicator bacteria showed that the resistance of the indicator bacteria was higher than that of the isolated strains. The presence of the genes *tet (L), tet (M), tet (O), tet (S), vanA, vanB*, and *Int1* was analyzed in the experiment, and neither P1 nor LY1 contained these genes. For LY2, only the *vanA* gene was detected.

Although these data illustrated the safety of the strains *in vitro*, we decided to also test the oral toxicity in animals to evaluate their safety *in vivo*. After being gavaged with the potential probiotic strains P1, LY1, and LY2 at high doses for 18 days, mice in P1 and LY1 presented no symptoms of illness, death or infection were recorded. However, LY2 was discarded due to vanA gene and poor mental state of the mice. Bacterial translocation is an important aspect of the detection of probiotic toxicity (Steffen and Berg, 1983). No bacteremia was recorded in any of the groups, and the detection rates of bacteria in the liver and kidney were similar between the control group and the experimental groups. Therefore, the occurrence of this translocation may not be related to the use of probiotics. In bacterial diseases prevention test in mice, P1 and LY1 were effective in reducing diarrhea associated with bacterial diseases. In conclusion, P1 and LY1 have strong antibacterial properties and probiotic potential and proved to be safe in the *in vivo* and *in vitro* experiments. Therefore, P1 and LY1 could be used to reduce diarrhea associated with bacterial diseases. Meanwhile, it probably has better clinical results if these two probiotics combined when administered because the two remaining strains have different antibacterial activities. Further experiments should be performed before these strains are used in clinical practice.

## Author Contributions

JL, YW, AL, and HZ provided the research idea. KM, XJ, MI, AL, JJ, MW, LZ, and YW contributed reagents, materials, and analysis tools. YW and AL wrote the manuscript. JL, HZ, and KM revised the manuscript. All authors participated in writing and reviewing the manuscript.

## Conflict of Interest Statement

The authors declare that the research was conducted in the absence of any commercial or financial relationships that could be construed as a potential conflict of interest.
