# Supplementary material for: Probiotic Potential of Leuconostoc pseudomesenteroides and Lactobacillus Strains Isolated From Yaks
Source: Front Microbiol. 2018 Dec 4;9:2987. doi: 10.3389/fmicb.2018.02987 (PMC6289064; doi:10.3389/fmicb.2018.02987)

**Table S1:** PCR primers used in this study

| **Target gene** | **Primer pair** | **5’–3’ Sequence** | **Reference** |
| --- | --- | --- | --- |
| *tet (L)*  *tet (M)*  *tet (O)*  *tet (S)*  *vanA*  *vanB*  *Int1* | *tet(L)-2-1*  *tet(L)-2-2*  *tet(M)-1*  *tet(M)-2*  *tet(O)-1*  *tet(O)-2*  *tet(S)-1*  *tet(S)-2*  *vanA1*  *vanA2*  *vanB for*  *vanB rev*  *Int1 (F)*  *Int1 (R)* | CATTTGGTCTTATTGGATCG  ATTACACTTCCGATTTCGG  GTTAAATAGTGTTCTTGGAG  CTAAGATATGGCTCTAACAA  GATGGCATACAGGCACAGAC  CAATATCACCAGAGCAGGCT  TGGAACGCCAGAGAGGTATT  ACATAGACAAGCCGTTGACC  GGGAAAACGACAATTGC  GTACAATGCGGCCGTTA  GTGCTGCGAGATACCACAGA  CGAACACCATGCAACATTTC  GGGTCAAGGATCTGGATTTCG  ACATGCGTGTAAATCATCGTCG | Aarestrup et al., 2000  Aarestrup et al., 2000  Aarestrup et al., 2000  Aarestrup et al., 2000  Dutkamalen et al.,1995  Ramos-Trujillo et al., 2003  Saenz et al., 2004 |

**Fig S1:** Photomicrographs of the liver of mice. (A) blank control group. (B) control group. (C) P1-2 group. (D) LY1-2 group. Bar= 50 um; H & E


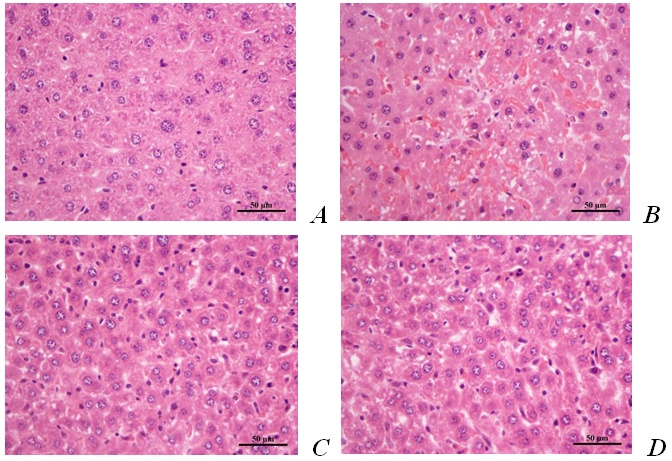


**Fig S2:** Photomicrographs of thespleen of mice. (A) blank control group.(B) control group. (C) P1-2 group. (D) LY1-2 group. Bar= 50 um; H & E


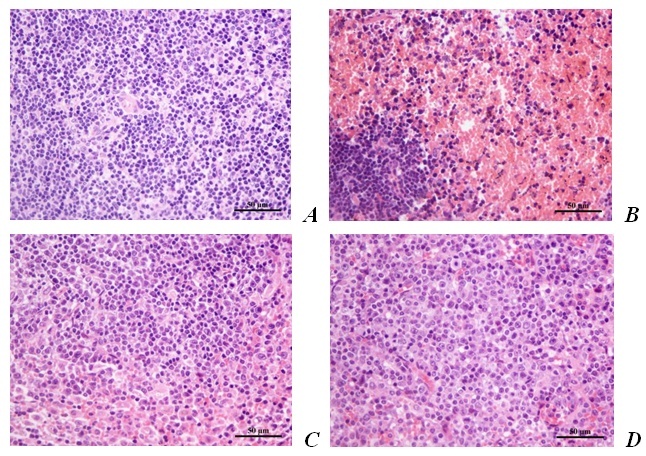


**Fig S3:** Photomicrographs of the duodenum of mice. (A) blank control group. (B) control group. (C) P1-2 group. (D) LY1-2 group. Bar= 100 um; H & E


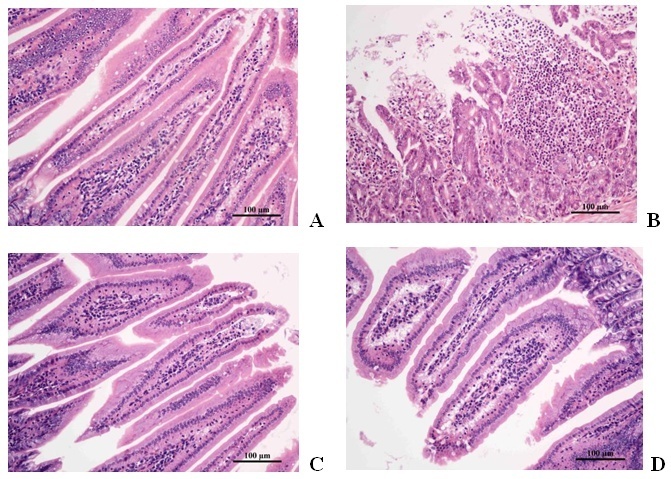

Supplement: Supplementary file 1 [file Data_Sheet_1.docx]
